# Role of Neuroactive Steroids in Health and Disease

**DOI:** 10.3390/biom14080941

**Published:** 2024-08-02

**Authors:** Roberto Cosimo Melcangi

**Affiliations:** Department of Pharmacological and Biomolecular Sciences “Rodolfo Paoletti”, Università degli Studi di Milano, 20133 Milan, Italy; roberto.melcangi@unimi.it

Steroidogenesis occurs not only in endocrine peripheral glands (i.e., gonads and adrenal glands) with the formation of steroid hormones but also in the nervous system through the synthesis of neurosteroids [1]. Indeed, cholesterol is converted by the mitochondrial enzyme P450 side-chain cleavage into pregnenolone, and then this steroid, via different enzymatic pathways, is converted into progesterone (PROG), androgens (e.g., deydroepiandrosterone, DHEA, and testosterone, T), estrogens (e.g., 17beta-estradiol, 17β-E), and corticosteroids (i.e., gluco- and mineralocorticoids) [2]. In addition, via the action of the enzymatic complex 5alpha-reductase and 3alpha- or 3beta-hydroxysteroid oxidoreductase, PROG and T may be converted into their subsequent metabolites, such as dihydroprogesterone, allopregnanolone (ALLO), and isoallopregnanolone (ISOALLO) in the case of PROG, and dihydrotestosterone, 3alpha-diol, and 3beta-diol in the case of T [3].

All of these molecules are included in the family of “neuroactive steroids” and regulate, through endocrine mechanisms (i.e., the steroid hormones) and/or paracrine and autocrine mechanisms (i.e., the neurosteroids), the neural functions [4]. These mechanisms are put into motion not only through interactions with classical steroid receptors (i.e., androgen, estrogen, glucorticoid, mineralocorticoid, and progesterone receptors) but also via membrane-bound receptors. For instance, PROG can also bind membrane receptors such as Sigma-1, membrane PRs (mPRs), and PR membrane components (PGRMCs) [5]. ALLO, as well as 3alpha-diol, can bind the gamma-aminobutyric acid (GABA)-A receptor [6,7], whereas the ALLO isomer, ISOALLO, does not bind to the GABA-A receptor but instead interferes with ALLO binding [8,9]. Moreover, 17β-E binds the G-protein-coupled ER 1 [10]. In addition, this steroid [11] and DHEA [12] may potentiate N-methyl-D-aspartate receptor activity. Neuroactive steroids exert a variety of physiological effects: for instance, they influence the regulation of memory, learning, myelination, reproductive behavior, and glial functions [13,14,15,16,17,18,19], as well as acting as protective agents in several neurodegenerative conditions (e.g., Alzheimer’s disease, Parkinson’s disease, multiple sclerosis, traumatic brain injury, stroke, and peripheral neuropathies) and psychiatric disorders (e.g., depression, anxiety, and post-traumatic stress) [2,20,21,22,23,24,25,26,27,28,29]. Interestingly, sex differences in the levels and actions of these molecules have been reported under physiological and pathological conditions [13,30,31]. Indeed, neuroactive steroids play a role in the sex differentiation of neural tissue during fetal development [32,33] and adult life [34,35]. Neurosteroidogenic enzymes and the related neuroactive steroid levels differ between the nervous systems of male and female rodents [2,36] and during the estrous cycle [37]. In addition, sex-specific alterations in the synthesis and levels of these molecules may occur during neuropathological events [2,38,39,40,41,42,43]. In agreement with this, some neuroactive steroids have been reported to exert sex-dimorphic neuroprotective effects in different animal models, suggesting a potential avenue for sex-targeted therapy based on neuroactive steroids [2,13,44,45]. 

With all of the aforementioned factors in mind, this Special Issue will discuss various aspects of neuroactive steroids and their use. For instance, as mentioned above, ALLO is a potent ligand of the GABA-A receptor; however, its function in the nucleus accumbens (a brain area associated with reward/motivation pathways) has been poorly explored. As discussed in this Special Issue by Mitchell et al. [46], this neurosteroid, via local synthesis, may play an important role in controlling GABA-ergic neurotransmission in this brain area, providing an important background for its use in postpartum depression. The importance of the local synthesis of neurosteroids, in terms of the altered metabolism of their substrate (i.e., cholesterol), is also discussed by Shu and collaborators [47]. Indeed, this manuscript reports on how the deletion of Cyp46a1 (an enzyme synthesizing the cholesterol oxidation product 24S-hydroxycholesterol) has an important impact on brain function. As mentioned above, altered patterns of neuroactive steroids are associated with neuropathological events. For instance, as discussed here in Diviccaro et al.’s study of an experimental model of type 2 diabetes mellitus, altered memory abilities are associated with a decrease in ALLO levels in the hippocampus, as well as neuroinflammation, oxidative stress, mitochondrial dysfunction, and altered gut microbiome composition [48]. In addition, Heckmann et al. explored the urinary levels of several steroids in preterm infants and observed that in early-preterm infants with the highest illness severity (<30 weeks), higher excretion rates of glucocorticoids and their precursors were associated with severe cerebral hemorrhage [49]. In this Special Issue, Cattane and collaborators report that in their study of an animal model of prenatal stress, as well as in an in vitro model of hippocampal progenitor cells treated with cortisol, miRNAs targeting FKBP5 (a stress-responsive gene involved in the effects of glucocorticoids), such as miR-20b-5p and miR-29c-3p, were significantly reduced, suggesting a key role for these miRNAs in sustaining the long-term effects of stress early in life [50]. As mentioned above, sex is another important variable. Indeed, a higher prevalence of Parkinson’s disease has been reported in men than in women [51,52], suggesting a possible role for estrogens in slowing the progression of this pathology. Furthermore, as reported by Lamontagne-Proulx et al. [53], mice overexpressing the human alpha-synuclein protein showed a more abrupt nigrostriatal dopamine decrease and an increase in microgliosis in males. However, at 18 months of age, sex differences were lost, probably because of the decrease in ovarian function [53]. As mentioned above, previous studies have also supported the protective role of neuroactive steroids. As reported here by Esperante and colleagues, treatment with T in an experimental model of amyotrophic lateral sclerosis (i.e., Wobbler mice) showed a variety of protective effects, including a reduction in myelin abnormalities, an improvement in motor performance, an enhancement of muscle mass, and an improvement in strength [54]. Also reported here, namely by Backstrom and colleagues, is that the progesterone metabolite ISOALLO which, as previously reported, acts as a GABA-A-modulating steroid antagonist [55,56] may inhibit estrus cycle-dependent aggressive behavior in rats [57]. Interestingly, similar observations regarding aggression or irritability and its linkage to the ovarian cycle are made in premenstrual dysphoric disorder [58]. As proposed in this Special Issue by Barreto [59], tibolone (a synthetic drug with estrogenic, androgenic, or progestogenic effects), actually used in clinics to treat menopause-related symptoms, could also be a therapeutic alternative for Alzheimer’s disease because of its ability to reduce amyloid burden and mitochondrial dysfunction.

Finally, as discussed here by Jevtovic-Todorovic and Todorovic [60], the capabilities of several steroids to interact with voltage-gated Ca^2+^ channels and GABA-A receptors suggest that neurosteroids represent a promising and safe new family of anesthetics.

Altogether, the manuscripts included in this Special Issue contribute exciting new discussions that highlight the important physiological and pathological roles of neuroactive steroids.

## References

[1] Corpechot C., Robel P., Axelson M., Sjovall J., Baulieu E.E. (1981). Characterization and measurement of dehydroepiandrosterone sulfate in rat brain. Proc. Natl. Acad. Sci. USA.

[2] Giatti S., Garcia-Segura L.M., Barreto G.E., Melcangi R.C. (2019). Neuroactive steroids, neurosteroidogenesis and sex. Prog. Neurobiol..

[3] Giatti S., Diviccaro S., Falvo E., Garcia-Segura L.M., Melcangi R.C. (2020). Physiopathological role of the enzymatic complex 5alpha-reductase and 3alpha/beta-hydroxysteroid oxidoreductase in the generation of progesterone and testosterone neuroactive metabolites. Front. Neuroendocrinol..

[4] Melcangi R.C., Garcia-Segura L.M., Mensah-Nyagan A.G. (2008). Neuroactive steroids: State of the art and new perspectives. Cell. Mol. Life Sci..

[5] Valadez-Cosmes P., Vazquez-Martinez E.R., Cerbon M., Camacho-Arroyo I. (2016). Membrane progesterone receptors in reproduction and cancer. Mol. Cell. Endocrinol..

[6] Lambert J.J., Belelli D., Hill-Venning C., Peters J.A. (1995). Neurosteroids and GABAA receptor function. Trends Pharmacol. Sci..

[7] Follesa P., Biggio F., Talani G., Murru L., Serra M., Sanna E., Biggio G. (2006). Neurosteroids, GABAA receptors, and ethanol dependence. Psychopharmacology.

[8] Hedstrom H., Bixo M., Nyberg S., Spigset O., Zingmark E., Backstrom T. (2009). Studies of pharmacokinetic and pharmacodynamic properties of isoallopregnanolone in healthy women. Psychopharmacology.

[9] Johansson M., Stromberg J., Ragagnin G., Doverskog M., Backstrom T. (2016). GABAA receptor modulating steroid antagonists (GAMSA) are functional in vivo. J. Steroid Biochem. Mol. Biol..

[10] Fuentes N., Silveyra P. (2019). Estrogen receptor signaling mechanisms. Adv. Protein Chem. Struct. Biol..

[11] Tauboll E., Sveberg L., Svalheim S. (2015). Interactions between hormones and epilepsy. Seizure.

[12] Bergeron R., de Montigny C., Debonnel G. (1996). Potentiation of neuronal NMDA response induced by dehydroepiandrosterone and its suppression by progesterone: Effects mediated via sigma receptors. J. Neurosci..

[13] Melcangi R.C., Giatti S., Garcia-Segura L.M. (2016). Levels and actions of neuroactive steroids in the nervous system under physiological and pathological conditions: Sex-specific features. Neurosci. Biobehav. Rev..

[14] Giatti S., Boraso M., Melcangi R., Viviani B. (2012). Neuroactive steroids, their metabolites and neuroinflammation. J. Mol. Endocrinol..

[15] Rasic-Markovic A., Djuric E., Skrijelj D., Bjekic-Macut J., Ignjatovic D., Sutulovic N., Hrncic D., Mladenovic D., Markovic A., Radenkovic S. (2024). Neuroactive steroids in the neuroendocrine control of food intake, metabolism, and reproduction. Endocrine.

[16] Melcangi R.C., Panzica G.C. (2014). Allopregnanolone: State of the art. Prog. Neurobiol..

[17] Garcia-Segura L.M., Mendez P., Arevalo M.A., Azcoitia I. (2023). Neuroestradiol and neuronal development: Not an exclusive male tale anymore. Front. Neuroendocrinol..

[18] Seib D.R., Tobiansky D.J., Meitzen J., Floresco S.B., Soma K.K. (2023). Neurosteroids and the mesocorticolimbic system. Neurosci. Biobehav. Rev..

[19] Walton N.L., Antonoudiou P., Maguire J.L. (2023). Neurosteroid influence on affective tone. Neurosci. Biobehav. Rev..

[20] Giatti S., Caruso D., Boraso M., Abbiati F., Ballarini E., Calabrese D., Pesaresi M., Rigolio R., Santos-Galindo M., Viviani B. (2012). Neuroprotective Effects of Progesterone in Chronic Experimental Autoimmune Encephalomyelitis. J. Neuroendocrinol..

[21] Giatti S., Mastrangelo R., D’Antonio M., Pesaresi M., Romano S., Diviccaro S., Caruso D., Mitro N., Melcangi R.C. (2018). Neuroactive steroids and diabetic complications in the nervous system. Front. Neuroendocrinol..

[22] Giatti S., Rigolio R., Romano S., Mitro N., Viviani B., Cavaletti G., Caruso D., Garcia-Segura L.M., Melcangi R.C. (2015). Dihydrotestosterone as a Protective Agent in Chronic Experimental Autoimmune Encephalomyelitis. Neuroendocrinology.

[23] Melcangi R.C., Giatti S., Pesaresi M., Calabrese D., Mitro N., Caruso D., Garcia-Segura L.M. (2011). Role of neuroactive steroids in the peripheral nervous system. Front. Endocrinol..

[24] Wang M., Hu S., Fu X., Zhou H., Yang S., Yang C. (2024). Neurosteroids: A potential target for neuropsychiatric disorders. J. Steroid Biochem. Mol. Biol..

[25] Morrow A.L., Boero G., Balan I. (2024). Emerging evidence for endogenous neurosteroid modulation of pro-inflammatory and anti-inflammatory pathways that impact neuropsychiatric disease. Neurosci. Biobehav. Rev..

[26] Peltier M.R., Verplaetse T.L., Altemus M., Zakiniaeiz Y., Ralevski E.A., Mineur Y.S., Gueorguieva R., Picciotto M.R., Cosgrove K.P., Petrakis I. (2024). The role of neurosteroids in posttraumatic stress disorder and alcohol use disorder: A review of 10 years of clinical literature and treatment implications. Front. Neuroendocrinol..

[27] Bourque M., Morissette M., Di Paolo T. (2024). Neuroactive steroids and Parkinson’s disease: Review of human and animal studies. Neurosci. Biobehav. Rev..

[28] Diviccaro S., Cioffi L., Falvo E., Giatti S., Melcangi R.C. (2021). Allopregnanolone: An overview on its synthesis and effects. J. Neuroendocrinol..

[29] Akwa Y. (2020). Steroids and Alzheimer’s Disease: Changes Associated with Pathology and Therapeutic Potential. Int. J. Mol. Sci..

[30] Giatti S., Diviccaro S., Serafini M.M., Caruso D., Garcia-Segura L.M., Viviani B., Melcangi R.C. (2020). Sex differences in steroid levels and steroidogenesis in the nervous system: Physiopathological role. Front. Neuroendocrinol..

[31] Melcangi R.C., Garcia-Segura L.M. (2010). Sex-specific therapeutic strategies based on neuroactive steroids: In search for innovative tools for neuroprotection. Horm. Behav..

[32] Cisternas C.D., Garcia-Segura L.M., Cambiasso M.J. (2017). Hormonal and genetic factors interact to control aromatase expression in the developing brain. J. Neuroendocrinol..

[33] Cisternas C.D., Tome K., Caeiro X.E., Dadam F.M., Garcia-Segura L.M., Cambiasso M.J. (2015). Sex chromosome complement determines sex differences in aromatase expression and regulation in the stria terminalis and anterior amygdala of the developing mouse brain. Mol. Cell. Endocrinol..

[34] Galea L.A.M., Frick K.M., Hampson E., Sohrabji F., Choleris E. (2017). Why estrogens matter for behavior and brain health. Neurosci. Biobehav. Rev..

[35] Cioffi L., Grassi D., Diviccaro S., Caruso D., Pinto-Benito D., Arevalo M.A., Garcia-Segura L.M., Melcangi R.C., Giatti S. (2024). Sex chromosome complement interacts with gonadal hormones in determining regional-specific neuroactive steroid levels in plasma, hippocampus, and hypothalamus. A study using the four core genotype mouse model. J. Steroid Biochem. Mol. Biol..

[36] Giatti S., Diviccaro S., Garcia-Segura L.M., Melcangi R.C. (2019). Sex differences in the brain expression of steroidogenic molecules under basal conditions and after gonadectomy. J. Neuroendocrinol..

[37] Cioffi L., Diviccaro S., Chrostek G., Caruso D., Garcia-Segura L.M., Melcangi R.C., Giatti S. (2024). Neuroactive steroids fluctuate with regional specificity in the central and peripheral nervous system across the rat estrous cycle. J. Steroid Biochem. Mol. Biol..

[38] Giatti S., D’Intino G., Maschi O., Pesaresi M., Garcia-Segura L.M., Calza L., Caruso D., Melcangi R.C. (2010). Acute experimental autoimmune encephalomyelitis induces sex dimorphic changes in neuroactive steroid levels. Neurochem. Int..

[39] Giatti S., Diviccaro S., Melcangi R.C. (2019). Neuroactive Steroids and Sex-Dimorphic Nervous Damage Induced by Diabetes Mellitus. Cell. Mol. Neurobiol..

[40] Giatti S., Rigolio R., Diviccaro S., Falvo E., Caruso D., Garcia-Segura L.M., Cavaletti G., Melcangi R.C. (2020). Sex dimorphism in an animal model of multiple sclerosis: Focus on pregnenolone synthesis. J. Steroid Biochem. Mol. Biol..

[41] Caruso D., D’Intino G., Giatti S., Maschi O., Pesaresi M., Calabrese D., Garcia-Segura L.M., Calza L., Melcangi R.C. (2010). Sex-dimorphic changes in neuroactive steroid levels after chronic experimental autoimmune encephalomyelitis. J. Neurochem..

[42] Pesaresi M., Giatti S., Cavaletti G., Abbiati F., Calabrese D., Bianchi R., Caruso D., Garcia-Segura L.M., Melcangi R.C. (2011). Sex differences in the manifestation of peripheral diabetic neuropathy in gonadectomized rats: A correlation with the levels of neuroactive steroids in the sciatic nerve. Exp. Neurol..

[43] Pesaresi M., Maschi O., Giatti S., Garcia-Segura L.M., Caruso D., Melcangi R.C. (2010). Sex differences in neuroactive steroid levels in the nervous system of diabetic and non-diabetic rats. Horm. Behav..

[44] Pesaresi M., Giatti S., Cavaletti G., Abbiati F., Calabrese D., Lombardi R., Bianchi R., Lauria G., Caruso D., Garcia-Segura L.M. (2011). Sex-dimorphic effects of dehydroepiandrosterone in diabetic neuropathy. Neuroscience.

[45] Peterson B.L., Won S., Geddes R.I., Sayeed I., Stein D.G. (2015). Sex-related differences in effects of progesterone following neonatal hypoxic brain injury. Behav. Brain Res..

[46] Mitchell S.J., Phillips G.D., Tench B., Li Y., Belelli D., Martin S.J., Swinny J.D., Kelly L., Atack J.R., Paradowski M. (2024). Neurosteroid Modulation of Synaptic and Extrasynaptic GABA(A) Receptors of the Mouse Nucleus Accumbens. Biomolecules.

[47] Shu H.J., Ziolkowski L.H., Salvatore S.V., Benz A.M., Wozniak D.F., Yuede C.M., Paul S.M., Zorumski C.F., Mennerick S. (2024). Effects of Complete and Partial Loss of the 24S-Hydroxycholesterol-Generating Enzyme Cyp46a1 on Behavior and Hippocampal Transcription in Mouse. Biomolecules.

[48] Diviccaro S., Cioffi L., Piazza R., Caruso D., Melcangi R.C., Giatti S. (2023). Neuroactive Steroid-Gut Microbiota Interaction in T2DM Diabetic Encephalopathy. Biomolecules.

[49] Heckmann M., Runkel A.S., Sunny D.E., Hartmann M.F., Ittermann T., Wudy S.A. (2024). Steroid Metabolomic Signature in Term and Preterm Infants. Biomolecules.

[50] Cattane N., Di Benedetto M.G., D’Aprile I., Riva M.A., Cattaneo A. (2024). Dissecting the Long-Term Effect of Stress Early in Life on FKBP5: The Role of miR-20b-5p and miR-29c-3p. Biomolecules.

[51] Moisan F., Kab S., Mohamed F., Canonico M., Le Guern M., Quintin C., Carcaillon L., Nicolau J., Duport N., Singh-Manoux A. (2016). Parkinson disease male-to-female ratios increase with age: French nationwide study and meta-analysis. J. Neurol. Neurosurg. Psychiatry.

[52] Taylor K.S., Cook J.A., Counsell C.E. (2007). Heterogeneity in male to female risk for Parkinson’s disease. J. Neurol. Neurosurg. Psychiatry.

[53] Lamontagne-Proulx J., Coulombe K., Morissette M., Rieux M., Calon F., Di Paolo T., Soulet D. (2023). Sex and Age Differences in a Progressive Synucleinopathy Mouse Model. Biomolecules.

[54] Esperante I.J., Meyer M., Banzan C., Kruse M.S., Lima A., Roig P., Guennoun R., Schumacher M., De Nicola A.F., Gonzalez Deniselle M.C. (2024). Testosterone Reduces Myelin Abnormalities in the Wobbler Mouse Model of Amyotrophic Lateral Sclerosis. Biomolecules.

[55] Lundgren P., Stromberg J., Backstrom T., Wang M. (2003). Allopregnanolone-stimulated GABA-mediated chloride ion flux is inhibited by 3beta-hydroxy-5alpha-pregnan-20-one (isoallopregnanolone). Brain Res..

[56] Backstrom T., Wahlstrom G., Wahlstrom K., Zhu D., Wang M.D. (2005). Isoallopregnanolone; an antagonist to the anaesthetic effect of allopregnanolone in male rats. Eur. J. Pharmacol..

[57] Backstrom T., Bengtsson S.K.S., Sjostedt J., Malinina E., Johansson M., Ragagnin G., Ekberg K., Lundgren P. (2023). Isoallopregnanolone Inhibits Estrus Cycle-Dependent Aggressive Behavior. Biomolecules.

[58] Sveinsdottir H., Backstrom T. (2000). Menstrual cycle symptom variation in a community sample of women using and not using oral contraceptives. Acta Obstet. Et Gynecol. Scand..

[59] Barreto G.E. (2023). Repurposing of Tibolone in Alzheimer’s Disease. Biomolecules.

[60] Jevtovic-Todorovic V., Todorovic S.M. (2023). The Role of Neuroactive Steroids in Analgesia and Anesthesia: An Interesting Comeback?. Biomolecules.

